# Trilogy of sequential infections in a diabetic male

**DOI:** 10.1002/rcr2.132

**Published:** 2015-11-18

**Authors:** Vikas Dogra, Deepak Talwar, Rajat Saxena, Charul Dabral, Sharad Joshi, Shobhit Bansal

**Affiliations:** ^1^ Metro Centre for Respiratory DiseasesMetro Multispeciality HospitalNoidaIndia

**Keywords:** Diabetes, klebsiella, mucormycosis, tuberculosis

## Abstract

Uncontrolled diabetes is a known immunosuppressive state. It predisposes individuals to bacterial and fungal infections. The present case report demonstrates sequential infections by Klebsiella followed by tuberculosis and later development of mucormycosis in a poorly controlled diabetic patient. Timing of diagnosis is of essence because of high mortality seen with such pulmonary infections. High index of suspicion needs to be maintained as the same individual may harbor multiple infections as highlighted in this case.

## Introduction

Diabetes is associated with depressed immune status. There is increased susceptibility to usual pneumonia as well as to opportunistic organisms like *Mucor* which normally do not produce disease in immunocompetent individuals despite their ubiquitous presence in the environment. Isolated case reports have shown co‐infection with *Mucor* and tuberculosis. The situation is particularly challenging when mucor infection develops while on treatment for tuberculosis as in our case report. Delays in diagnosis can increase mortality exponentially in mucormycosis. As a consequence a high index of suspicion, prompt diagnostic evaluation is necessary.

## Case Report

**Figure 1:**
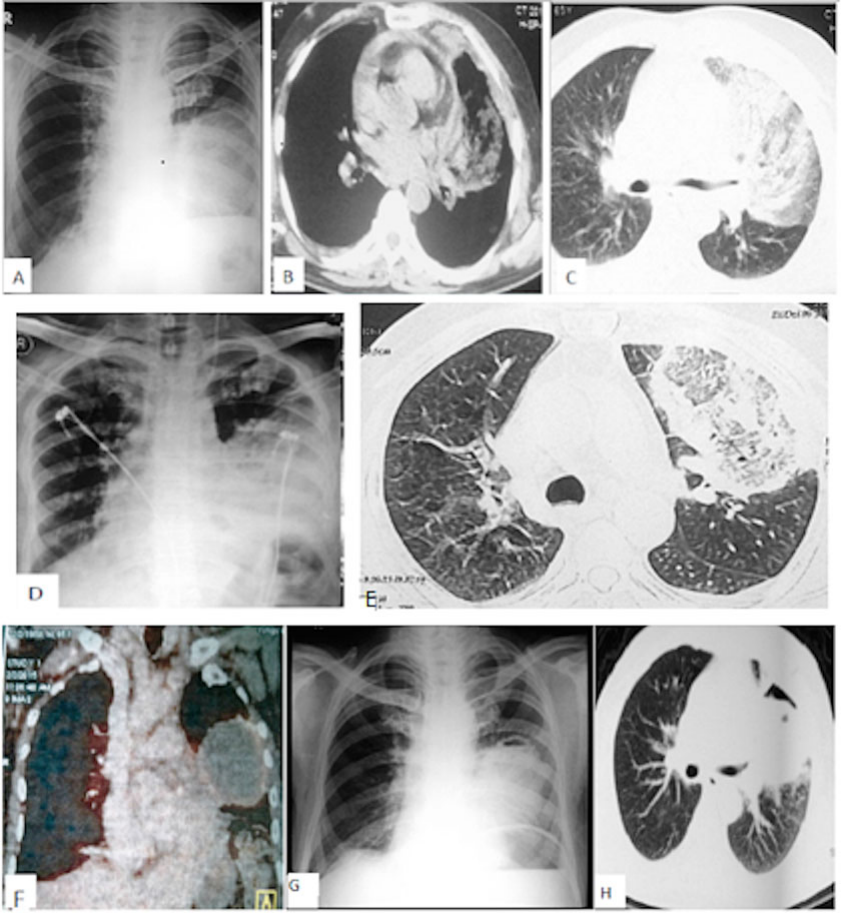
CXR‐PA and CT scan of the thorax showing left upper lobe consolidation (A–C), left upper lobe consolidation with reverse halo sign (D,E), PET CT scan (F) and air crescent sign (G,H).

A 68‐year‐old male presented to the emergency room with left‐sided pleuritic chest pain, productive cough, fever, and progressive breathlessness of 20‐day duration. He was a known diabetic on oral hypoglycemic agents for 20 years. A chest radiograph and computed tomography (CT) scan of the thorax performed prior to admission showed a left upper lobe consolidation (Fig. 1A,B,C). Sputum culture done at that time revealed growth of an extended spectrum beta lactamase producing *Klebsiella pneumoniae*. The patient was treated with parenteral Meropenem for two weeks. On arrival in the emergency department he was tachypneic with a peripheral blood oxygen saturation on room air of 86%. Laboratory investigations revealed evidence of diabetic ketoacidosis (Blood sugar‐316 mg/dl, urine ketones ++, Glycosylated hemoglobin [HbA1C]‐9.70), as well as anemia (hemoglobin [Hb]‐8.9 g/dl) and leucocytosis (14,800/mm3, 84% neutrophils). Repeat CT thorax showed a left upper lobe consolidation with a reverse halo sign (Fig. 1D,E). Common causes of reverse halo sign are enumerated in Table 1 .

**Table 1:** Causes of reverse halo sign

Cryptogenic organizing pneumonia	Pneumocystis pneumonia
Opportunistic invasive fungal infections (IFI)	Tuberculosis
Pulmonary mucormycosis	Community‐acquired pneumonia
Invasive pulmonary aspergillosis (IPA)	Lymphomatoid granulomatosis
Paracoccidioidomycosis	Lipoid pneumonitis
Granulomatosis with polyangiitis	Pulmonary neoplasms
Sarcoidosis	Pulmonary infarction
	Radiation therapy and radiofrequency/microwave ablation of pulmonary malignancies

Empiric voriconazole therapy was commenced because of suspicion of a fungal pneumonia based on the imaging findings. Bronchoalveolar lavage (BAL) staining demonstrated acid fast bacilli. No fungal hyphae were seen. BAL galactomannan was negative. Endobronchial biopsy and CT guided Tru‐cut biopsy of the consolidated area revealed no acid‐fast bacilli or fungal elements. Patient's blood sugar level was optimized. Antituberculous therapy was started based on the BAL report. The patient showed marked clinical improvement over the next four months. However, consolidation persisted on chest imaging. Repeat CT thorax revealed an air crescent sign which prompted repeat diagnostic evaluation (Fig. 1G,H). Positron emission tomography‐CT revealed a well defined area of consolidation with air bronchograms and features of necrosis. There was mild peripheral fluorodeoxyglucose (FDG) uptake in left upper lobe and lingula with few subcentimetric mediastinal and left mammary lymph nodes (Fig. 1F).

**Figure 2:**
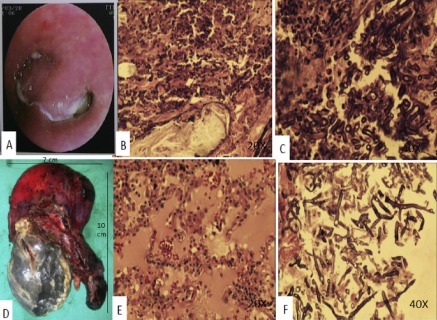
Marked narrowing of the left upper lobe bronchus (A), neutrophilic infiltrate and eosinophils with broad‐based obtuse angle branching aseptate fungal hyphae (B,C), resected left upper lobe (gross) (D), alveoli filled with PAS positive proteinaceous material (E) and necrotic foci with mucor (F).

Fiberoptic bronchoscopic examination showed a marked narrowing of the left upper lobe bronchus (Fig. 2A). Endobronchial biopsy demonstrated broad‐based obtuse angle branching aseptate fungal hyphae suggestive of mucormycosis (Fig. 2B,C).

Liposomal amphotericin B was initiated and a left upper lobectomy planned. The patient was not given clearance in the pre‐anesthetic check‐up because of deranged thyroid functions. A left upper lobectomy was done after three weeks when these normalized (Fig. 2D).

Cut section of the resected specimen revealed gray‐white areas with necrotic foci. Microscopy demonstrated alveoli filled with periodic acid‐Schiff positive proteinaceous material suggestive of secondary pulmonary alveolar proteinosis. Hyphae suggestive of mucormycosis were also seen (Fig. 2E,F).

An increase in serum creatinine on amphotericin B prompted a change in antifungal therapy to posaconazole which the patient was discharged on, together with his antituberculous treatment. At six months, the patient is doing well and remains asymptomatic with good glucose control and normal renal functions.

## Discussion

Poorly controlled diabetes mellitus with or without diabetic ketoacidosis is also a known predisposing factor for mucormycosis in 36%–88% of cases [1].

Mucormycosis refers to several different opportunistic diseases caused by infection with fungi in the order of Mucorales. They are saprophytic fungi ubiquitous in soil and decaying organic material. *Rhizopus* species are the most common causative organisms followed by *Mucor*. A hallmark of mucormycosis is extensive angio‐invasion with resultant vessel thrombosis and tissue necrosis.

The overall mortality rate in patients with pulmonary mucormycosis is high, partly because of delayed diagnosis.

An observational study previously reported apparent distinguishing features in patients with pulmonary disease caused by *Aspergillus* species as compared to zygomycetes. Peripheral nodular lesions appeared to be more indicative of zygomycosis, with mass lesions, cavitation, consolidation, and a halo sign being more indicative of *Aspergillus* infection [2]. In our case, the CT findings were more suggestive of an *Aspergillus* infections.

In a case control study of 27 cases of zygomycosis, associated risk factors were noted to be voriconazole use, malnutrition, and diabetes mellitus [3]. Our patient had all three risk factors.

A rare finding in this case was the presence of pulmonary alveolar proteinosis secondary to *mucor* infection. A case of disseminated *mucor* with pulmonary alveolar proteinosis in a patient with leukemia was previously reported in 1960 [4]. Secondary PAP has been associated with many conditions (Table 2).

**Table 2:** Causes of secondary PAP

Immunodeficiency	Hematological disorders
Severe combined immunodeficiency	Myelodysplastic syndromes
Agammaglobulinemia	Acute myeloid leukemia
Pulmonary pneumocystosis secondary to HIV infection	Acute lymphoid leukemia
Organ transplantation	Lymphoma
Connective tissue diseases	Myeloma
Dermatomyositis	Non‐hematological cancers
Rheumatoid arthritis	Squamous cell cancer
Behcet's disease	Adenocarcinoma
Inhalation of toxic particles	Mesothelioma
Silica, talc, cement, kaolin	Melanoma with lung metastasis
Metal particles (aluminum, titanium, indium)	Glioblastoma
Organic particles (fibers of cellulose)	

In patients with pulmonary mucormycosis, surgical treatment in conjunction with antifungal therapy has been shown to significantly improve survival when compared with antifungal therapy alone. Surgical debridement of infected tissue should be performed on an urgent basis.

Optimal therapy is uncertain. Currently, the recommended antifungal therapy for zygomycosis includes amphotericin B at the highest tolerated dosage, usually 1.0 to 1.5 mg/kg per day. Posaconazole possesses potent activity against mucor and is now increasingly being used for prophylaxis in immunosuppressed patients.

There have been isolated case reports of coinfection with tuberculosis and mucormycosis [5]. This case shows progression of mucormycosis in a patient diagnosed with tuberculosis in spite of there being no evidence of mucor in initial diagnostic samples. This case illustrates the importance of maintaining constant vigilance in high‐risk individuals such that an early diagnosis of mucormycosis can be made and outcomes are improved.

## Disclosure Statements

No conflict of interest declared.

Appropriate written informed consent was obtained for publication of this case report and accompanying images.
